# Comparing the Effectiveness of Intravenous Tissue Plasminogen Activator and Dual Antiplatelet Therapy in Patients With Minor Stroke: A Meta-Analysis

**DOI:** 10.7759/cureus.46436

**Published:** 2023-10-03

**Authors:** Obinna Monday, Anurag Rawat, Abraham K Isaak, Amima Manzoor, Goldi Jaiswal, Leena Saeed, Ajanta Kumari, Adil Amin

**Affiliations:** 1 Medicine, Norfolk and Norwich University Hospital, Norwich, GBR; 2 Interventional Cardiology, Himalayan Institute of Medical Sciences, Dehradun, IND; 3 Telemetry, Sharp Memorial Hospital, San Diego, USA; 4 Internal Medicine, Orotta School of Medicine and Dentistry, Asmara, ERI; 5 Medicine, Jinnah Sindh Medical University, Karachi, PAK; 6 Medicine, Manipal College of Medical Sciences, Pokhara, NPL; 7 Internal Medicine, National Ribat University, Khartoum, SDN; 8 Internal Medicine, Dow University of Health Sciences, Karachi, PAK; 9 Cardiology, Pakistan Navy Ship Shifa (PNS Shifa), Karachi, PAK

**Keywords:** systematic review and meta-analysis, dual antiplatelet therapy, effectiveness, stroke, intravenous tissue plasminogen activator

## Abstract

The aim of this study was to compare the outcomes between dual antiplatelet therapy (DAPT) versus intravenous tissue plasminogen activator (IV t-PA) in patients with minor stroke. This meta-analysis follows the Preferred Reporting Items for Systematic Reviews and Meta-analyses (PRISMA) reporting guidelines. Two authors independently conducted online database searches using PubMed, Web of Science, and EMBASE to identify articles published in English language from inception to September 5, 2023. Outcomes assessed in this meta-analysis included all-cause mortality, stroke incidence, and functional outcomes (measured by modified ranking scale (mRS) scores of 0 to 1). A total of three studies fulfilled the eligibility criteria and included in the final analysis. Pooled analysis showed that the risk of all-cause mortality was not significantly different between the t-PA group and DAPT group (relative risk (RR): 1.14, 95% confidence interval (CI): 0.32-4.06). Compared with those treated with DAPT, there was no significant difference in t-PA in terms of the number of patients with a favorable functional outcome (defined as an mRS score of 0-1). The risk of stroke was not significantly different between the t-PA group and DAPT group (RR: 1.11, 95% CI: 0.68 to 1.82). The analysis, based on three studies, revealed no significant differences between t-PA and DAPT regarding all-cause mortality, stroke incidence, and functional outcomes.

## Introduction and background

Acute ischemic stroke (AIS) is a prevalent condition associated with significant morbidity, mortality, and disability [1]. Recent estimates suggest that there are approximately three million new cases of stroke each year, with around 30% classified as minor strokes [2]. Unfortunately, only 44% of individuals experiencing acute minor strokes or transient ischemic attacks (TIAs) receive treatment within three hours of symptom onset [3]. The term “minor stroke” is often used for stroke patients with mild and nondisabling symptoms. However, a consensus definition is lacking [2]. Within three months of their initial stroke, roughly 10% to 20% of patients experience a subsequent stroke, with half of these occurring within two days [4]. However, available strategies to prevent these high recurrence rates are limited. The main strategy employed is dual antiplatelet therapy (DAPT). Research, such as the findings from the Clopidogrel with Aspirin in Acute Minor Stroke or Transient Ischemic Attack (CHANCE) study and the Platelet-Oriented Inhibition in New TIA and Minor Ischemic Stroke (POINT) trial, has indicated that combining clopidogrel with aspirin can lead to a decrease in the risk of another stroke by 32.0% and 27%, respectively [5-6].

The use of intravenous tissue plasminogen activator (IV t-PA) as a valid treatment for AIS, especially in cases of minor stroke, remains a subject of controversy [7]. A lack of sufficient evidence regarding the effectiveness of IV thrombolysis in patients with minor strokes is a key factor contributing to this uncertainty. Many randomized controlled trials (RCTs) have excluded individuals with minor strokes from their studies [8-9]. A prior study suggested that 30% of patients with minor strokes who did not receive IV t-PA treatment may experience poor outcomes [10]. The 2018 guidelines for AIS suggest that IV t-PA should be contemplated as a treatment option for individuals experiencing minor strokes with disabling symptoms within three hours of the onset of symptoms. In addition, it may be deemed reasonable for those with minor strokes occurring within the 3- to 4.5-hour timeframe [11].

DAPT typically combines aspirin with a P2Y12 receptor inhibitor (e.g., clopidogrel or ticagrelor). This regimen works by preventing platelet aggregation and reducing the risk of blood clot formation [12]. T-PA, on the other hand, is a thrombolytic agent that aids in dissolving blood clots by converting plasminogen into plasmin, an enzyme responsible for breaking down fibrin, the primary component of blood clots [13]. Currently, there is limited research comparing DAPT with t-PA. Consequently, this study has been undertaken to conduct a comprehensive pooled analysis of available, with the aim of assessing the effectiveness of DAPT versus t-PA.

## Review

Methodology

This meta-analysis follows the Preferred Reporting Items for Systematic Reviews and Meta-analyses (PRISMA) reporting guidelines. The meta-analysis was registered with PROSPERO (International Prospective Register of Systematic Reviews) (registration no. CRD42023447521).

Search Strategy and Literature Search

Two authors independently conducted online database searches using PubMed, Web of Science, and EMBASE to identify articles published in English from inception up to September 5, 2023. The search strategy incorporated key terms, such as "dual antiplatelet therapy," "intravenous tissue plasminogen activator," and "stroke" along with their synonyms and medical subject heading (MeSH) terms (Appendix 1). Besides this, we used boolean algebra operators to further sensitize the search. Then, the reference lists of all included articles were manually reviewed to identify any additional studies relevant to the study's topic.

Study Selection

Two authors independently screened titles and abstracts following the initial search. After eliminating duplicate entries, the initial screening was conducted based on abstracts and titles, followed by a full-text screening process using detailed inclusion and exclusion criteria. Any disagreements in the study selection process were resolved through a consultation with the principal investigator.

We included all studies that met the following criteria: (1) observational studies or RCTs comparing recombinant tissue-type plasminogen activator (r-tPA) and DAPT in patients with mild stroke and (2) studies reporting the required outcomes. We excluded studies that lacked a comparison group, as well as case reports, case series, reviews, and editorials.

Data Extraction and Outcomes

Two authors performed data extraction using a pre-designed data extraction form created with Microsoft Excel. They subsequently cross-verified their extracted data to reach a consensus. Data extraction covered various aspects, including study details, such as the primary author's name, whether it was a single-center or multi-center study, study design, follow-up period, sample size, and the geographical region where it was conducted. In addition, we extracted patient characteristics, including age and gender. Outcomes assessed in this meta-analysis included all-cause mortality, stroke incidence, and functional outcomes (measured by modified ranking scale (mRS) scores of 0 to 1).

Data Analysis

We conducted data analysis using ReviewManager (RevMan) version 5.4.1 from the Cochrane Collaboration. Outcomes were reported as risk ratios (RRs) with 95% confidence intervals (CI). P-values less than 0.05 were considered statistically significant. We assessed statistical heterogeneity using the I^2^ statistic. When heterogeneity was not statistically significant (I^2^ ≤ 50%), we applied the fixed-effects model. In cases where significant heterogeneity was present among study results (I^2^ > 50%), we utilized the random-effects model. 

Results

**Figure 1:**
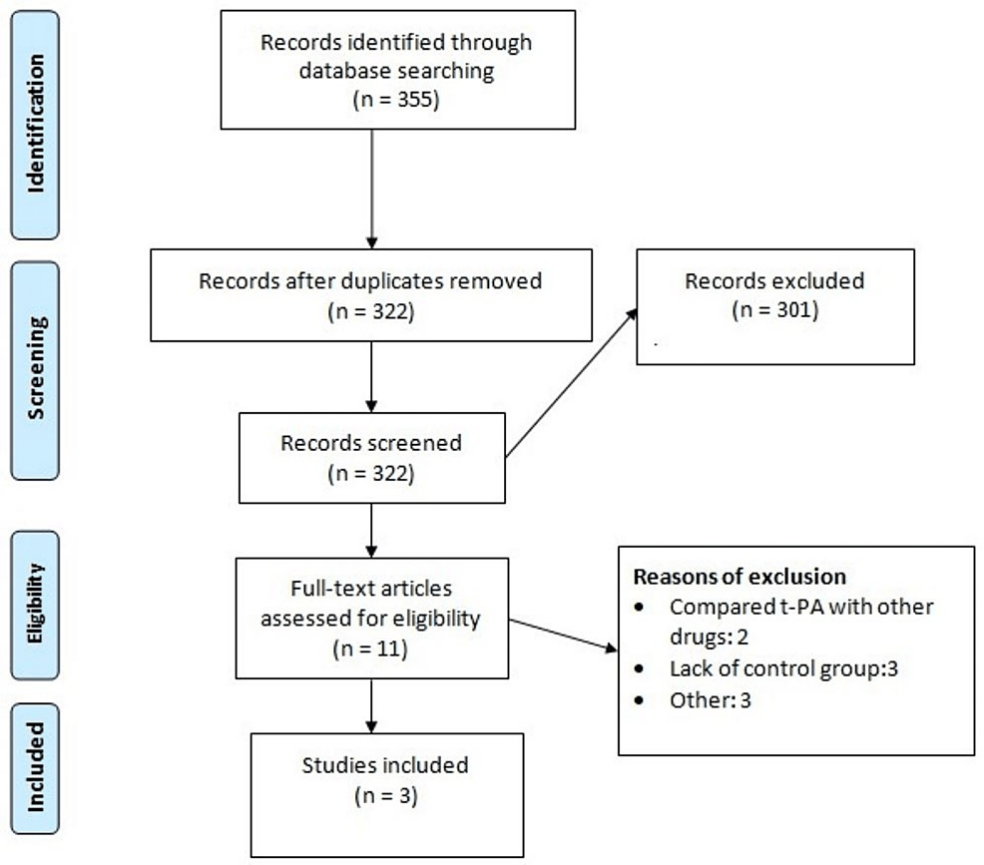
PRISMA flowchart of the selection of studies PRISMA: Preferred Reporting Items for Systematic Reviews and Meta-analyses

Figure 1 shows the PRISMA flowchart of the selection of studies. Online database searching yielded 355 articles. After removing 33 duplicate articles, 322 studies were initially screened using titles and abstracts. A detailed assessment of 11 studies was done using predefined inclusion and exclusion criteria. Finally, three studies fulfilled the eligibility criteria and included in the final analysis. Table 1 shows the characteristics of the included studies. Table 1 shows the characteristics of the included studies. Out of three included studies, two were retrospective cohort and one was an RCT. Majority of the participants in all the included studies were males.

**Table 1:** Characteristics of the included studies RCT: randomized controlled trial; RC: retrospective cohort; DAPT: dual antiplatelet therapy, t-PA: tissue plasminogen activator

Study ID	Year	Study design	Setting	Region	Groups	Sample size	Follow-up	Mean age (years)	Males (n)	Hypertension (n)	Diabetes (n)
Chen et al. [14]	2023	RCT	Multicenter	China	t-PA	350	90 days	64	240	169	86
					DAPT	369		65	256	211	101
Duan et al. [15]	2022	RC	Single center	China	t-PA	251	90 days	62	178	166	52
					DAPT	722		63	510	468	211
Wang et al. [16]	2021	RC	Multicenter	China	t-PA	385	90 days	61	257	252	77
					DAPT	215		63.8	149	135	52

All-Cause Mortality

**Figure 2:**
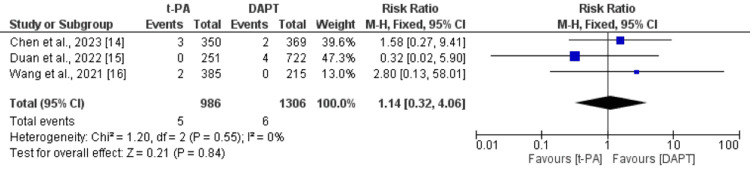
Comparison of all-cause mortality between the t-PA and DAPT groups DAPT: dual antiplatelet therapy, t-PA: tissue plasminogen activator Sources: references [14-16]

All three studies reported all-cause mortality. As shown in Figure 2, the risk of all-cause mortality was not significantly different between the t-PA group and DAPT group (RR: 1.14, 95% CI: 0.32-4.06). No significant heterogeneity was reported among the study results (I^2^: 0%).

Functional Outcomes

**Figure 3:**
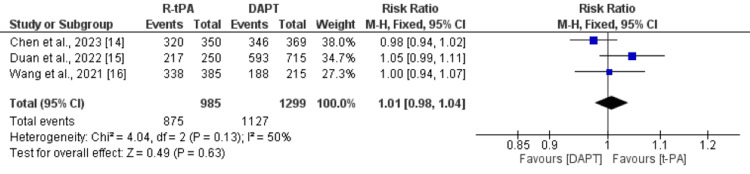
Comparison of functional outcomes (mRS score 0 to 1) between the DAPT and t-PA groups DAPT: dual antiplatelet therapy, t-PA: tissue plasminogen activator; mRS: modified ranking scale Sources: references [14-16]

**Figure 4:**
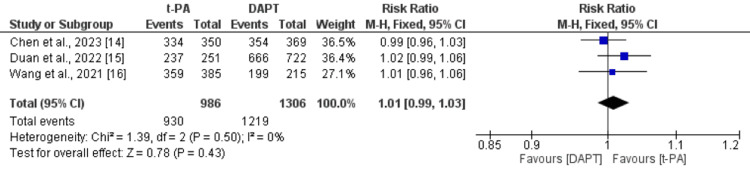
Comparison of functional outcomes (mRS 0-2) between the t-PA and DAPT groups DAPT: dual antiplatelet therapy; t-PA: tissue plasminogen activator Sources: references [14-16]

Compared with those treated with DAPT, there was no significant difference in t-PA in terms of the number of patients with favorable functional outcome (defined as an mRS score of 0 to 1), as shown in Figure 3. Similarly, the number of patients with an mRS score of 0-2 was also not significantly different between the patients treated with DAPT and t-PA, as shown in Figure 4.

Stroke Recurrence

**Figure 5:**
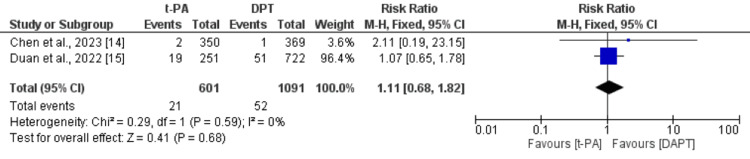
Comparison of the incidence of stroke between the t-PA and DAPT groups DAPT: dual antiplatelet therapy; t-PA: tissue plasminogen activator Sources: references [14-15]

As shown in Figure 5, the pooled incidence of stroke was 4.31%. The risk of stroke was not significantly different between the t-PA group and DAPT group (RR: 1.11, 95% CI: 0.68 to 1.82). No significant heterogeneity was reported among the study results (I^2^: 0%).

Discussion

Our meta-analysis has examined the efficacy outcomes of t-PA and DAPT in individuals with mild stroke. We did not find any significant differences between t-PA and DAPT in terms of all-cause mortality, stroke incidence, and functional outcomes. It is essential to note that this appears to be the first meta-analysis comparing the effectiveness of t-PA and DAPT specifically in patients with mild stroke. However, given the relatively limited number of studies available for analysis, we must exercise caution in interpreting these findings, and additional research is needed to draw definitive conclusions regarding whether t-PA is non-superior or superior to DAPT.

Previous research has provided valuable insights into the effectiveness and safety of DAPT for individuals who have experienced minor strokes. For example, the Fast Assessment of Stroke and Transient Ischemic Attack to Prevent Early Recurrence (FASTER) trial demonstrated that administering a 300 mg loading dose of clopidogrel within 24 hours of symptom onset, followed by daily aspirin at 75 mg, could potentially reduce the risk of stroke without increasing the likelihood of intracranial hemorrhage [17]. Subsequently, the CHANCE trial, a meticulously designed study, reported that the early and short-term use of a combination of clopidogrel and aspirin was significantly more effective in reducing the risk of recurrent ischemic stroke when compared to aspirin alone in patients who had experienced a minor stroke [5].

A study conducted by Duan et al. compared patients receiving t-PA with those in a non-t-PA group and found that IV t-PA was associated with favorable functional outcomes at discharge, although no significant difference in all-cause mortality was observed [18]. While IV t-PA is regarded as one of the most effective treatments for individuals with acute ischemic stroke, it is generally not recommended for those with minor strokes [19]. According to the Chinese National Stroke Registry (CNSR), a substantial proportion of patients (41.8% out of 2,514) who arrived at the emergency department within three hours of symptom onset did not receive IV t-PA treatment primarily because their symptoms were mild and rapidly improved [20]. Although the National Institute of Neurological Disorders and Stroke (NINDS) rt-PA Stroke Study did not identify a significant difference in the beneficial effects of IV t-PA in patients with minor strokes compared to those who received a placebo, there was an observed trend suggesting that minor stroke patients who received IV t-PA experienced favorable outcomes [21]. In our study, 90% of the patients in both the t-PA group and DAPT group achieved good functional outcomes, defined as mRS scores of 0 to 1. However, there was a slightly higher proportion in the t-PA group, although the difference was not statistically significant.

Our study revealed a subtle trend, although not statistically significant, in the three-month functional outcomes among patients with acute minor stroke who received IV t-PA treatment, DAPT, and aspirin. However, it is important to recognize that IV t-PA treatment may be a potential option for managing certain individuals with minor stroke, particularly those who experience early neurological deterioration (END). The occurrence of END varied between 10% and 40% and was associated with unfavorable short-term and long-term results [22]. Even though minor stroke symptoms were mild and brain lesions were small in many cases, a portion of patients still experienced END [23]. Findings from the CT/CT angiography and MRI-based "CATCH" study indicated that not only stroke recurrence but also the progression of stroke contributed to poor outcomes in individuals with minor stroke. Furthermore, stroke progression was identified as an independent risk factor for an unfavorable prognosis at 90 days [24].

DAPT may be effective in preventing END, but recent research discovered that its effectiveness depended on whether the patient carried cytochrome P450 2C19 loss-of-function alleles, with positive outcomes in carriers and no significant effect in non-carriers [25]. Notably, minor stroke cases involving intracranial artery occlusion were linked to severe END, and IV t-PA treatment appeared to reduce the risk of non-hemorrhagic END [26]. Therefore, determining the optimal approach to treating acute minor stroke necessitates a comprehensive assessment of each individual's condition.

The latest discoveries underscore the importance of understanding the societal benefits associated with enhancing our knowledge regarding the outcomes associated with IV t-PA therapy in patients with minor strokes [27]. To achieve this objective, conducting well-designed RCTs, larger-scale investigations, and real-world research initiatives is imperative for a comprehensive evaluation of the effectiveness and safety of IV t-PA treatment in individuals with minor strokes.

In our study, although the overall rate of stroke within 90 days was higher in patients receiving IV t-PA than in the DAPT group, the rate of stroke was comparable, with no significant difference observed between the two groups. However, future studies are needed to validate these findings and determine whether DAPT and t-PA are effective treatments for patients with minor stroke.

Study Limitations

The present meta-analysis has several limitations. First, it incorporated only three studies, and among these, just one was an RCT. Consequently, further research is warranted to elucidate the role of t-PA in patients with minor stroke more comprehensively. Second, because we lacked individual-level data, conducting subgroup analyses was not possible. Third, all the studies evaluated outcomes at the 90-day mark. Consequently, there is a need for future studies that compare the long-term outcomes of these drugs in individuals with minor stroke.

## Conclusions

Our meta-analysis compared the effectiveness of t-PA and DAPT in patients with mild stroke. The analysis, based on three studies, revealed no significant differences between t-PA and DAPT regarding all-cause mortality, stroke incidence, and functional outcomes. The current meta-analysis provides valuable insights into the treatment options for mild stroke, but further research is necessary to confirm the role of t-PA and to explore long-term outcomes. The management of acute minor stroke requires careful consideration, and the optimal approach may vary depending on individual patient characteristics. Future well-designed clinical trials and larger-scale studies will be crucial in advancing our understanding of the outcomes associated with IV t-PA treatment in patients with minor stroke and in determining the most effective treatment strategies for this patient population.

## Appendices

PubMed

“dual antiplatelet therapy” [MeSH Terms] OR “DAPT” [All fields] AND “intravenous tissue plasminogen activator” [All fields] OR “t-PA” [MeSH Terms] OR “alteplase” [All fields] OR “reteplase” [All fields] OR “tenecteplase” [All fields] AND “stroke” [MeSH Terms] OR “minor stroke” [All fields] OR “mini stroke” [All fields] OR “transient ischemic attack” [All fields] OR “TIA” [All fields]

Web of Science

TS=("dual antiplatelet therapy" OR DAPT) AND ("intravenous tissue plasminogen activator" OR t-PA OR alteplase OR reteplase OR tenecteplase) AND ("stroke" OR "minor stroke" OR "mini stroke" OR "transient ischemic attack" OR TIA))

This search strategy uses the following syntax:

·         TS = Topic Search

·         " = quotation marks to search for exact phrases

·         OR = Boolean operator to combine search terms

·         AND = Boolean operator to require all search terms to be present in a document

EMBASE

"dual antiplatelet therapy" OR "DAPT" AND "intravenous tissue plasminogen activator" OR "tPA" OR "alteplase" OR "reteplase" OR "tenecteplase" AND ("stroke" OR "minor stroke" OR "mini stroke" OR "transient ischemic attack" OR "TIA")

This search strategy uses the following syntax:

·         AND = Boolean operator to require all search terms to be present in a document

·         OR = Boolean operator to combine search terms

·         " = quotation marks to search for exact phrases
